# Lessons from Chlorophylls: Modifications of Porphyrinoids Towards Optimized Solar Energy Conversion

**DOI:** 10.3390/molecules191015938

**Published:** 2014-10-03

**Authors:** Dariusz Karcz, Bożena Boroń, Arkadiusz Matwijczuk, Justyna Furso, Jakub Staroń, Alicja Ratuszna, Leszek Fiedor

**Affiliations:** 1 Faculty of Biochemistry, Biophysics and Biotechnology, Jagiellonian University, Gronostajowa 7, 30-387 Kraków, Poland; E-Mails: dariusz.karcz@uj.edu.pl (D.K.); bozenaboron@wp.pl (B.B.); arkadiusz.matwijczuk@up.lublin.pl (A.M.); justyna.furso@gmail.com (J.F.); jakubstaron@gmail.com (J.S.); 2 A Chełkowski Institute of Physics, University of Silesia, Uniwersytecka 4, 40-007 Katowice, Poland; E-Mail: alicja.ratuszna@us.edu.pl; 3 Department of Biophysics, University of Life Sciences in Lublin, Akademicka 13, 20-950 Lublin, Poland; 4 Institute of Pharmacology, Polish Academy of Sciences, Smętna 12, 31-343 Kraków, Poland

**Keywords:** singlet excited state lifetime, quantum yield, pigment photostability, pigment aggregation, central metal ion, molecular symmetry

## Abstract

Practical applications of photosynthesis-inspired processes depend on a thorough understanding of the structures and physiochemical features of pigment molecules such as chlorophylls and bacteriochlorophylls. Consequently, the major structural features of these pigments have been systematically examined as to how they influence the S_1_ state energy, lifetimes, quantum yields, and pigment photostability. In particular, the effects of the macrocyclic π-electron system, central metal ion (CMI), peripheral substituents, and pigment aggregation, on these critical parameters are discussed. The results obtained confirm that the π-electron system of the chromophore has the greatest influence on the light energy conversion capacity of porphyrinoids. Its modifications lead to changes in molecular symmetry, which determine the energy levels of frontier orbitals and hence affect the S_1_ state properties. In the case of bacteriochlorophylls aggregation can also strongly decrease the S_1_ energy. The CMI may be considered as another influential structural feature which only moderately influences the ground-state properties of bacteriochlorophylls but strongly affects the singlet excited-state. An introduction of CMIs heavier than Mg^2+^ significantly improves pigments' photostabilities, however, at the expense of S_1_ state lifetime. Modifications of the peripheral substituents may also influence the S_1_ energy, and pigments’ redox potentials, which in turn influence their photostability.

## 1. Introduction

The ever growing demand for energy sources other than conventional fossil fuels is a strong stimulus for studies on artificial solar energy conversion [1,2,3,4]. Solar technologies rely on conversion of photons into a more usable form of energy such as electricity, heat or chemical energy. One of the approaches for the development of artificial photosynthetic devices involves an engineering of solar energy-converting materials with properties that mimic those of natural photosynthetic pigments. The light energy conversion in dye solar cells begins with photon capture, which requires chromophores with strong absorption of light in the UV-Vis region. The photon absorption leads to the promotion of electrons in the dye valence shell and subsequent conversion of the excitation energy into charge separation, which must occur with minimal energy losses due to dissipational pathways. To cope with this constant energy flow a good thermal- and photostability of the dye in both its ground- and excited-states is necessary.

**Figure 1:**
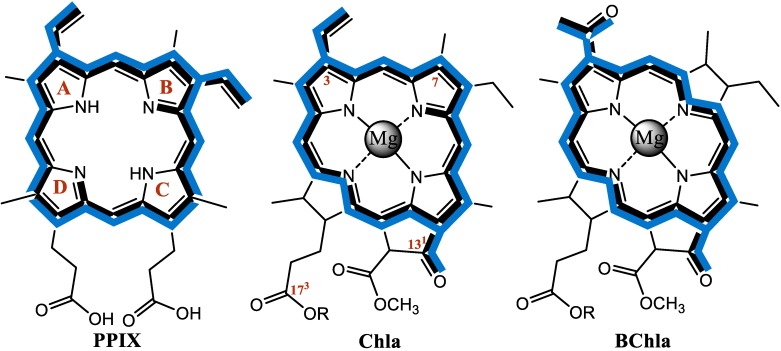
Structural formulae of PPIX, Chla, and BChla with blue color representing the conjugated π-electron system. The numbering system of the pyrrolenine rings is shown at PPIX, and the numbering of the carbon skeleton is shown at Chla.

In natural photosynthetic systems these criteria are fulfilled best by (bacterio)chlorophylls ((B)Chls) embedded in the protein matrix. These chromophores are tetrapyrrole-derived macrocycles, in which four nitrogen atoms form the central metal binding pocket, usually occupied by a Mg^2+^ ion. The periphery of the chlorophyll (Chl) and bacteriochlorophyll (BChl) macrocycle is functionalized with two main conservative moieties, namely, a fused five member isocyclic ring and a long-chain aliphatic alcohol (Figure 1). As the periphery of Chls can be functionalized with a variety of additional substituents, over 50 naturally occurring chlorophyllous structures have been reported to date [5,6,7,8,9]. These structural features determine the ground- and excited-state properties of Chls, essential for efficient solar energy conversion. As Chls and BChls play a major role in all the primary steps of photosynthesis, *i.e.* light-harvesting, charge separation and electron transfer across the photosynthetic membranes, the understanding of mechanisms underlying their functioning and their functional versatility has been a subject of intensive studies for more than a century [6,10]. Various aspects of their photochemistry relevant to photosensitization and reactions involving a reactive oxygen species (ROS), have also been investigated [11] and became of interest since photodynamic therapy (PDT) emerged as a promising cancer treatment method [12,13].

Practical applications of photosynthesis-inspired processes depend on a thorough understanding of the structures and physiochemical features of Chls. The main aim of this work is to evaluate the feasible modifications of Chls and assess their effects on the key features of these pigments in the context of tuning pigment capacity for solar energy conversion outside the photosynthetic apparatus. In particular, we address a question of how these modifications influence the following parameters considered critical to solar energy conversion: the S_1_ state energy, its lifetime (τ_fl_), and quantum yield (Φ_fl_), and pigment photostability in solution.

Indeed, the literature describing the ground- and excited-state properties of porphyrins, (B)Chls and their derivatives is rich (see leading reviews) [6,7,14,15,16]. However, a direct comparison between various pigments, such as porphyrins and (B)Chls, may be somewhat difficult because the original data were obtained using different pigment isolation methodologies and different instrumentation, also in terms of technological advancement. Often, the measurements were carried out under non-equivalent conditions, which precludes any meaningful comparative analysis in search of fine structural effects. In this context, only a few reports exist in which a range of pigments are investigated under strictly the same conditions, including their isolation and handling, and hardware. Moreover, in terms of systematic structural relationships, some earlier reports, focusing on particular classes of pigments, seem fragmentary and may require verification using modern instrumentation. Therefore, the aim of the present work is to assess both the gross and subtle differences between closely related chromophores, attempting to minimize the discrepancies in pigment isolation/handling and hardware. The effects of modifications were investigated in the following series of pigments: protoporphyrin IX (PPIX), chlorophyll a (Chla), chlorophyll b (Chlb), and bacteriochlorophyll a (BChla), their free bases; pheophytin a (Pheo), bacteriopheophytin a (BPheo), and the Zn-, Ni-, and Pt-substituted derivatives of Chla (Zn-Pheo, Ni-Pheo, and Pt-Pheo, respectively). The ground- and excited-state properties of the model pigments (their structures are shown in Figure 1) were examined using a range of spectroscopic methods, including electronic absorption, circular dichroism (CD), steady state and time-resolved emission spectroscopy.

## 2. Results and Discussion

### 2.1. Results

#### 2.1.1. Ground- and Excited-State Properties

**Figure 2:**
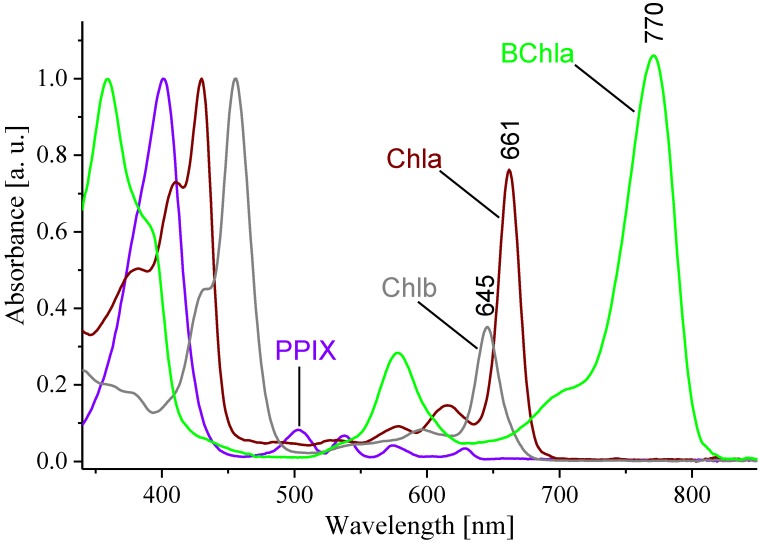
Electronic absorption spectra of PPIX, Chla, Chlb, and BChla recorded at ambient temperature in acetone. The spectra were normalized to the maxima of the Soret bands.

In the absorption spectrum of PPIX (Figure 2), a typical porphyrin, the maximum of the Soret band is located at 400 nm and there are four low intensity Q bands in the 480–650 nm region. The Chls have their Soret band (S_3_ level) maxima shifted to 400–500 nm and their strong Q_Y_ bands (S_1_ level) are positioned between 600 and 700 nm.

**Figure 3:**
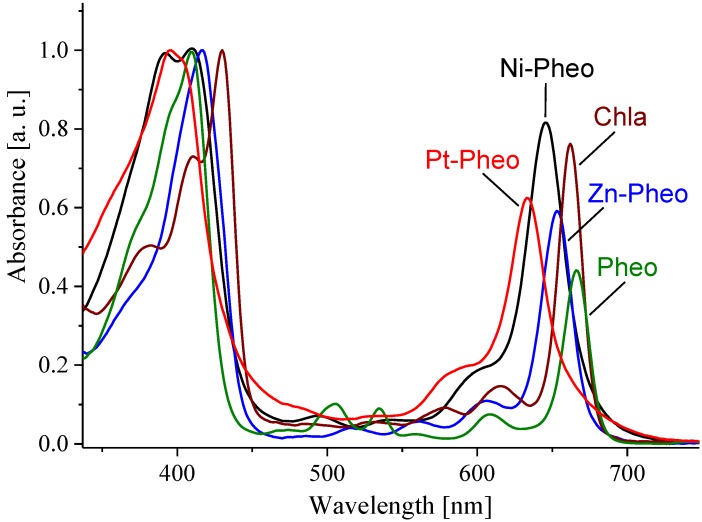
Electronic absorption spectra of Pheo and its complexes with Mg^2+^, Zn^2+^, Ni^2+^, and Pt^2+^, recorded at ambient temperature in acetone. The spectra were normalized to the Soret bands’ maxima.

The BChl derivatives have their Soret band maxima positioned between 340 and 400 nm, while intensive Q_X_ and Q_Y_ maxima lie between 500‒600 nm and 700‒800 nm, respectively. In these pigments, the Q_X_ band (S_2_ level) is much better resolved than in Chls and PPIX. The ratio between the intensities of the Soret and Q_Y_ bands is the highest for Chlb, and the lowest for BChla, while the corresponding ratio for Chla is intermediate. Figure 3 depicts the central metal ion (CMI) effects on the electronic transitions of metallo-substituted Chls. The Q_Y_ maxima of the complexes are in the range of 600‒700 nm and their intensities vary depending on the inserted metal ion. The Q_X_ bands are not well-resolved and only minor shifts of the Soret bands are observed. The Q_Y_ maximum in Pt-Pheo shows the largest blue shift compared to that of Pheo.

**Figure 4:**
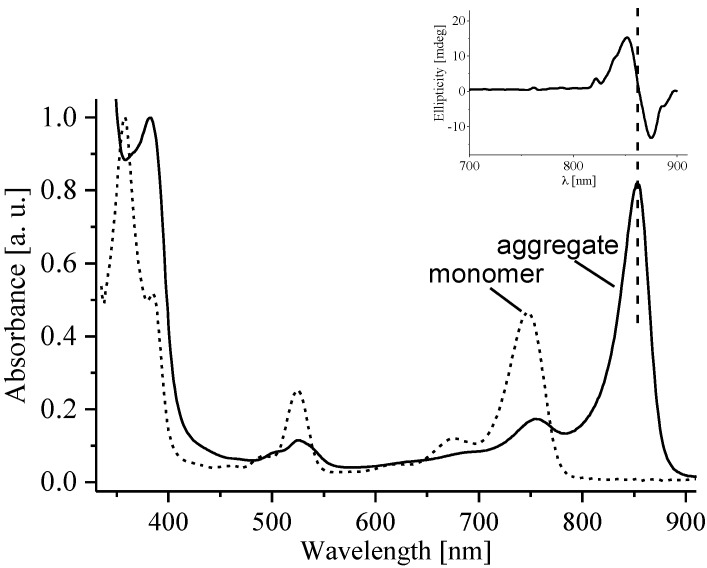
Electronic absorption spectra of monomeric (in acetone) and aggregated BPheo in β-OG micellar system. In the insert, the circular dichroism spectrum of the aggregated form is shown. All the measurements were performed in 1 cm quartz cells at ambient temperature.

The absorption spectrum of monomeric BPheo was recorded in acetone and compared with that of BPheo aggregate in a micellar system (Figure 4). The absorption spectra of monomeric and aggregated BChla were measured in a similar manner (not shown). Due to aggregation, a new transition appears on the lower energy side of the monomeric band with the maximum at 750 nm. The BPheo aggregate emits fluorescence of a very low intensity (fl_max_ = 890 nm, not shown) and its Φ_fl_ value is negligible. The CD spectra of the BPheo aggregates show strong Cotton effects in the Soret and Q_Y_ regions (Figure 4), while no such effects are observed for the monomeric forms.

The exact energies of the S_0_→S_1_ transition in the model pigments, collected in Table 1, were determined from the intersections of the absorption and fluorescence spectra, which both were normalized to ~1 at the Q_Y_ and fluorescence maxima (fl_max_), respectively. In comparison to the free bases, the S_1_ energies of Chls are higher, while an opposite effect is observed in BChla. The presence of Zn^2+^, Ni^2+^ or Pt^2+^ as the CMIs in Pheo causes some shifts in the S_1_ energy with respect to the Mg^2+^ complex (Table 1).

**Table 1:** Selected ground- and excited-state properties of model pigments estimated in acetone at ambient temperature.

Pigment	S_1_ energy [cm^−1^] (± 2%)	τ_fl_ [ns] (± 2%)	Φ_fl_ (± 5%)
PPIX	15833	11.2	0.03
Chla	15025	5.7	0.27
Chlb	15401	3.7	0.20
BChla	12758	2.9	0.23
Pheo	14924	6.6	0.27
BPheo	13234	2.4	0.09
Zn-Pheo	15216	3.3	0.13
Ni-Pheo	15218 ^a^	nd	nd
Pt-Pheo	15205 ^a^	nd	nd

nd—not determined, ^a^—Q_Y_ maximum.

The central Ni^2+^ or Pt^2+^ ions cause a complete shut off of emission while the other pigments are fluorescent and their fluorescence emission decays are monoexponential (not shown). As seen in Table 1, the longest S_1_ state lifetime, as inferred from the τ_fl_ values, is observed in PPIX (11.2 ns), shorter in Chls (5–7 ns), and the shortest in BChls (~2.6 ns). The removal of the central Mg^2+^ ion from Chla results in a longer S_1_ state lifetime (6.6 ns) while the insertion of Zn^2+^ shortens the S_1_ state lifetime (3.3 ns). The values collected in Table 1 are consistent with the ones reported previously [17,18,19,20,21,22,23].

The Φ_fl_ values, directly related to S_1_ quantum yield, were estimated using Equation (1), based on the parameters obtained from the absorption and fluorescence spectra [24,25]:

(1) $${\text{Φ}}_{fl}={\text{Φ}}_{fl}^{0}\cdot \frac{\int fl(\lambda )d\lambda }{\int fl_{0}(\lambda )d\lambda }\cdot \frac{A_{0}}{A}\cdot \frac{n^{2}}{n_{0}^{2}}$$

in which ${\text{Φ}}_{fl}^{0}$ is a standard fluorescence quantum yield of Chla in MeOH ∫fl(λ)dλ is a surface area under the fluorescence spectrum, *n* represents the solvents refractive index, and A is the absorbance at the excitation wavelength. The subscript 0 refers to Chla used as standard. The absorbance of each sample was kept at 0.15 at the Q_Y_ maximum. Chla and Pheo show the highest (0.27) and PPIX the lowest (0.03) Φ_fl_ values. The intermediate values were found for BChla, Chlb, Zn-Pheo, and BPheo (0.23, 0.20, 0.13, and 0.09, respectively). The insertion of Zn^2+^ ion into Pheo results in an almost 50% decrease in Φ_fl_ compared to that of Chla. All the investigated ground- and excited-state parameters are very similar to those previously published for the individual pigments [18,20,26].

#### 2.1.2. Pigment Photostability

The photostability of pigments in EtOH was assessed based on the time during which a 50% decay of their Q_Y_ bands occurs (τ_1/2_). The photostability of monomers in micellar system was determined in a similar manner, while the assessment of half-lifetimes of aggregates was based on the decay of their lowest energy band. The photostability studies were carried out in conditions that enable direct comparisons of the outcomes (see the Experimental Section).

**Figure 5:**
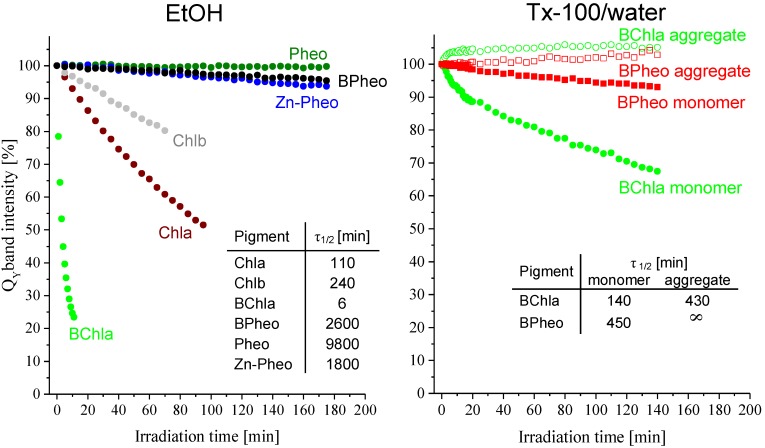
Photostabilites of Chla, Chlb, BChla, Pheo, and Zn-Pheo in EtOH (left), and of the BChla and BPheo aggregates in TX-100/water (right). The photostabilities of pigments are expressed as the decay of the lowest energy bands upon irradiation with red light (see text for details). Pigment half-lifetime (τ_1/2_) was estimated as the time during which a 50% decay of the Q_Y_ band occurs.

The photostabilities in EtOH order the pigments as follows: Pheo > BPheo > Zn-Pheo > Chlb > Chla >> BChla (Figure 5). Pheo is the most (τ_1/2_ = 9,800 min) and BChla least photostable pigment (τ_1/2_ = 6 min) in the series. Zn-Pheo is fairly photostable (τ_1/2_ = 1,800 min), while Chlb and Chla are significantly less photostable (τ_1/2_ of 240 and 110 min, respectively).

In the micellar system, the BPheo monomer is more photostable than BChla (τ_1/2_=450 and 140 min, respectively, Figure 5). The τ_1/2_ of BChla aggregate was estimated as 430 min, which is comparable to that of BPheo monomer. No photodegradation of the BPheo aggregate was detected, which indicates that the BPheo aggregate is far more photostable than that of BChla.

### 2.2. Discussion

#### 2.2.1. Model System

The selection of model pigments was done on the basis of their availability and chemical stability as well as their relevance to photosynthesis [6]. Chla, Chlb and BChla are the most abundant and relatively stable chlorin/bacteriochlorin type molecules and together with their corresponding free bases may serve as convenient model pigments. The peripheral carbonyl groups in Chlb (C7) and in BChla (C3) make these molecules particularly interesting. Thus, in our model series, the structures change from porphyrins through chlorin to bacteriochlorin (Figure 1). Also the occupancy of the central cavity of Pheo is modified with metal ions, *i.e.*, Mg^2+^, Zn^2+^, Ni^2+^, and Pt^2+^, and the macrocycle periphery is substituted with various functional groups. The commercially available and chemically stable PPIX was used as a representative possessing the porphyrin skeleton. Below, the effects of these modifications are discussed in detail.

#### 2.2.2. Symmetry of the Macrocyclic π-Electron System

The S_1_ energies, the S_1_ state lifetime and its quantum yield strongly depend on the structure and symmetry of the π-electron system of the pigments. In a highly symmetrical PPIX the S_1_ energy is the highest (~15,830 cm^−1^), while in the homologues of lower symmetry, Pheo and BPheo, it progressively decreases (~14,920, and ~13,230 cm^−1^, respectively). Thus, the S_1_ energies change in the following order: porphyrins>>chlorins>bacteriochlorins (Table 1), which is well established in the literature [14,26]. If the aggregation is taken into account, the S_1_ energy is lower than that in the monomer due to the excitonic coupling, which is reflected in the absorption spectra of aggregates by the appearance of a new, low energy absorption band. In BPheo aggregate the S_1_ energy is ~11,740 cm^−1^ (Figure 4), and even lower in BChla (~12,020 cm^−1^, not shown). The occurrence of excitonic interactions is confirmed by Cotton effects present in the CD spectra of BPheo (Figure 4 inset) and BChla aggregates (not shown), consistent with the literature [27]. In effect, the S_1_ energy, due to structural modifications and/or aggregation can be lowered along the series by approximately 4,100 cm^−1^, with respect to PPIX.

If the free bases are considered, the Φ_fl_ values in Pheo and BPheo (0.27 and 0.09, respectively) are several fold higher than that in PPIX (0.03), consistent with previously published results [17,20,26]. Each step of symmetry lowering in the free bases results in the shortening of S_1_ state lifetime by approximately a factor of two, from 11.2 ns in PPIX, to 6.6 ns in Pheo and 2.4 ns in BPheo. Similar trends are observed in Chla and BChla, with their respective S_1_ state lifetime values of 5.7 and 2.9 ns. The shortening of S_1_ state lifetime values in chlorins and bacteriochlorins is also related to their molecular symmetry, and is consistent with the energy gap law, which in molecules of low symmetry postulates an overlap of oscillatory levels of the S_0_ and S_1_ state that favors the non-radiative relaxation. Secondly, it reflects the fact that more intense absorption is correlated with the shortening of the transition lifetime.

Obviously, the size and shape of the π-electron system, are the major determinants of the optical properties of (B)Chls. The differences in molecular symmetries of porphyrins, chlorins and bacteriochlorins reflect on the energies of their frontier orbitals, and hence affect all the ground- and excited-state properties of these compounds as shown in this and many previous studies [28,29]. Especially, the S_1_ energies closely correspond to the molecular symmetry and the macrocycle saturation. The order of S_1_ energies (porphyrins>>chlorins>bacteriochlorins) is consistent with the widely accepted Gouterman’s model, which links it with a molecular symmetry-dependent size of the energy gap between HOMO and LUMO [30]. Additionally, the S_1_ energies may also be affected by the central metal ion and the peripheral substituents (see below).

The macrocyclic π-electron system in (B)Chls also participates in a series of stacking interactions which govern the aggregation and thus affect the ground- and excited-state properties of these pigments. The π-π stacking is stronger in the free bases, while in the Mg complexes it is somewhat weaker, likely due to steric effects caused by axial ligation. The emission of aggregates is either low or non-detectable, which may be caused by light scattering [31] or other emission quenching mechanism [32].

#### 2.2.3. Type of Central Metal Ion

The metalation of Pheo results in a blue shift of up to 800 cm^−1^ in the Q_Y_ band, which is more than in the BChla series (500 cm^−1^) [33]. The S_1_ energy increases and the S_1_ state lifetime shortens with the increased atomic mass of CMI. The emission lifetime is the longest in Pheo (6.6 ns) and shortens to 5.7 and 3.3 ns in the Mg-, and Zn-complex, respectively, while in Ni-Pheo and Pt-Pheo it remains below the detection level. The Φ_fl_ values for Pheo and Chla are the same (0.27) within the experimental error (Table 1). The insertion of Zn^2+^ causes a 50% decrease in Φ_fl_, while in Ni-Pheo and Pt-Pheo it could not be determined due to an extremely low signal (Table 1). The Ni^2+^ ion in Ni-Pheo is responsible for an extremely rapid, radiationless decay of the excited state S_1_ [23]. The metalation with yet heavier metal ions, such as Pt^2+^, introduces a strong spin-orbit coupling due to heavy metal effect, which favors the relaxation *via* intersystem crossing (ISC) [34,35], and increases the yield of the T_1_ state [22].

The CMI also affects the higher excited states of the pigments, *i.e.*, the Soret and even more significantly the Q_X_ transition. Also the intensities of Q_Y_ vary depending on the CMI (Figure 3). These changes are related to axial ligation, and are both CMI- and solvent-dependent [33,36]. The CMI, by introducing an electron-withdrawing center, particularly strongly affects the energy levels of orbitals with high electron densities localized on core N atoms [36,37]. Also the CMI binding energy increases parallel with CMI electronegativity [38]. The metal-core interactions in the metallocomplexes change from relatively weak electrostatic in Chla to stronger classical coordination bonds in Zn-Pheo, and strong coordination-covalent interactions in Ni-Pheo [39]. Both the shortening of S_1_ state lifetime and the decrease in Φ_fl_ seem correlated with the increasing strength of CMI bonding in the central pocket along the Mg-Zn-Ni/Pt series, resulting in an efficient intracomplex quenching of the chromophore excited state. In effect, the redox potentials and electronic transition energies also change [21], and so does pigment photostability.

#### 2.2.4.Peripheral Substituents

Among the peripheral substituents present in Chls the carbonyl groups conjugated to the macrocyclic π-electron system, *i.e.* the C7 in Chlb, and C3 in BChla, are the most noteworthy. These groups belong to auxochromes and hence affect the S_1_ energies, but the extent of this influence is rather subtle compared to that of the core π-electron system. A clear effect of the C7 carbonyl is seen in Chlb, in which it brings about a 370 cm^−1^ blue shift of the S_1_ energy compared to that of Chla. An even more significant effect of this group is seen in the S_1_ state lifetimes, which shorten from 5.7 ns in Chla to 3.7 ns in Chlb. In BChla, the S_1_ state lifetime is even shorter, but the effect caused by this group may overlap with that of the symmetry change. Although the presence of peripheral carbonyl groups may influence on Φ_fl_, this parameter is governed mostly by the macrocyclic π-electron system and CMI type.

Other peripheral substituents also may affect the ground- and excited-state properties. The C3 functionality is directly attached to the conjugated π-system, and hence modifications at this position have the strongest influence on the optical properties of the chromophore. Secondly, as the C3 atom is positioned near the y-molecular axis, the most significant changes concern the S_1_ energy. The C7 substituent interacts with the chromophore system only in chlorins, while in bacteriochlorins it is separated from the conjugated π-system. Since the C7 position lies near the x-molecular axis, it mainly influences on the S_2_ state, which is of minor importance in the context of solar energy conversion. The phytyl (C17^3^) does not seem to have much influence on the π-electron system, but in fact this bulky residue indirectly affects (B)Chls properties, mostly *via* the steric effects, and thus the solvation of macrocycle [19,21,40].

#### 2.2.5. Isocyclic Ring

This conservative structural feature of Chls certainly affects their ground- and excited-state properties, but the extent of this influence is difficult to define. Seemingly, the most significant changes are associated with a decrease in molecular symmetry and an increase in macrocycle rigidity in (B)Chls, compared to that of porphyrins. Secondly, the S_1_ energy in (B)Chls is related to the electronic transitions along the molecular y-axis, which passes through the N atoms of rings A and C. As the isocyclic ring is fused with the ring C, it influences the S_1_ energy of the pigments, probably by extending the system of conjugated bonds towards the C13^1^ carbonyl. In aggregation of (B)Chls in organic solvents, the isocyclic ring with the carbonyl moiety at the 13^1^-position is a site of intermolecular interactions, *e.g*. hydrogen bonds [41].

#### 2.2.6. Pigment Photostability

The successive saturation of the macrocycle is accompanied by changes in redox behavior of the pigments [42]. Particularly, the oxidation potentials decrease from porphyrins to chlorins and bacteriochlorins [7]. This trend corresponds with their photostabilities [43], especially in the Mg^2+^ complexes, where BChla, with its lower oxidation potential [44] than that of Chla or Chlb, is much less photostable. An analogical trend is observed in the free bases, but the differences are less pronounced. The insertion of metal ions heavier than Mg^2+^ leads to an enhancement of photostability, as a comparison of Chla *vs.* Zn-Pheo shows (Figure 5). Interestingly, the photostability of Zn-Pheo is still lower than that of Pheo. Ni-Pheo demonstrates an exceptional photostability (almost no photodegradation) [23].

The peripheral substituents, such as the C7-carbonyl, seem beneficial for photostability, since Chlb is notably more photoresistant than Chla. On the other hand, the very low photostability of BChla may be associated with the presence of C3-carbonyl. This comparison shows that several factors, such as peripheral substituents, CMI, hydrogen bonding, and even the distortions of the macrocycle affect individual redox potentials, and thus the photostabilities [37,43,44].

Aggregation also increases the photostabilities of pigments and it is clear that the aggregate structure, which is governed by the presence/lack of the central Mg^2+^ ion, affects the photostabilities. The characteristic fluorescence quenching in the aggregates (not shown) suggests that the relaxation from the S_1_ state occurs *via* either the radiationless mechanism or generation of the triplet state [45,46,47]. Seemingly, the exceptional photostability of BPheo aggregate may be related to the strong π-π stacking which allows for an efficient radiationless (e.g., vibrational) dissipation of the absorbed energy. In the BChla aggregate the strength of these π-π interactions is much lower, probably due to the steric hindrance caused by the CMI and axial ligands, consequence of which is a more efficient generation of the triplet state. In the presence of oxygen it may lead to the degradation of BChla molecules.

## 3. Experimental Section

### 3.1. Materials

All solvents were of 99% purity or higher (HPLC grade), and were used without further purification, except acetic acid (POCh, Gliwice, Poland), which was doubly distilled prior to use. Methanol (MeOH), 2-propanol (IPA), acetone, acetonitrile (ACN) and *n-*hexane were purchased from Rathburn Chemicals (Walkeburn, Scotland). Ethanol (EtOH) was purchased from Merck (Darmstadt, Germany). PPIX, formic acid and gels for column chromatography were purchased from Sigma (Poznań, Poland).

### 3.2. Methods

#### 3.2.1. Isolation and Purification of Pigments

Chla and Chlb were extracted from spinach leaves [48] and separated by column chromatography, using Sepharose CL-6B as the stationary phase and 1.5% IPA in *n-*hexane to elute Chla, and 10% IPA in *n-*hexane to elute Chlb. BChla was isolated from the cells of purple photosynthetic bacteria *Rhodobacter sphaeroides* and purified by column chromatography on DEAE-Sepharose CL-6B, using 10% MeOH in acetone as the eluent [23,49]. The demetalation of Chla and BChla in glacial acetic acid yielded the corresponding free bases, Pheo and BPheo, further purified by column chromatography; Pheo on Sepharose CL-6B, and BPheo on DEAE Sepharose CL-6B. All pigments were finally purified by isocratic HPLC, using a ProStar 200 (Varian, Santa Clara, CA, USA) apparatus equipped with a diode detector 1024 VIS TIDAS (J&M, Essingen, Germany). The pigment separations were carried out on a RP-C18 column (250 × 4.6 mm, Varian) using a mixture of MeOH‒ACN‒IPA (45:45:10 v/v) at the flow rate of 2 mL/min, except the metallo-substituted derivatives. These separation conditions allowed for a direct comparison of the retention times of the pigments (see Supplementary Materials). Fractions containing allomers and epimers were not collected.

The syntheses and purification of Zn-Pheo, Pt-Pheo, and Ni-Pheo, were carried out according to previously published protocols [23,33,50]. The identity of isolated pigments was confirmed by mass spectrometry using an ACQUITY TQD mass spectrometer (Waters, Milford, MA, USA) with an electrospray positive ionisation method. The spectrometer was coupled with an ACQUITY H-Class UPLC apparatus (Waters) equipped with an ACQUITY UPLC column (BEH C18 1.7 μm, 2.1 × 50 mm, Waters). MeOH acidified with 0.1% formic acid was used as eluent (see Supplementary Materials). All synthetic and purification steps were carried out under dim light and the isolated pigments were stored in the dark, at −30 °C, under Ar.

#### 3.2.2. Pigment Aggregation

The aggregation was carried out by adding an aliquot (10 μL) of pigment stock solution in acetone to a 1 cm path length quartz cuvette filled with detergent solution (1.5 mL), while stirring. A 0.8% and 2% solution (w/v) of *n*-octyl–β–d–glucopyranoside (β-OG) in 20 mM Tris-HCl buffer, pH = 7.8 (TB) was used for the aggregation of BChla and BPheo, respectively. The mixture was stirred for 15 min on a magnetic stirrer and portions of pigment were added in 15 min intervals until the absorption spectrum indicated the formation of aggregates, and then until the absorbance at the lowest energy band of aggregate was in the range of 0.6–0.8. The final concentration of acetone in each mixture did not exceed 10% (v/v). Triton (Tx-100) was used in the photostability studies on monomeric BPheo and BChla in micellar system.

#### 3.2.3. Photostability Study

The pigments dissolved in EtOH to give an absorbance of ~0.5 at the Q_Y_ maximum were illuminated using a halogen light source (Schott KL 1500, LCD, Mainz, Germany) equipped with fiber optics. The beam of white light was passed through a water layer (heat filter) and then through a red cut-off filter RG 630 (Schott, λ ≥ 630 nm). The photodegradation of BPheo and BChla aggregates in micellar systems was examined using the same methodology, except that 855 nm bandpass filter (Carl Zeiss, Oberkochen, Germany) was used for the illumination of aggregates, while the BChla and BPheo monomers were illuminated using 775 and 752 nm bandpass filters (Carl Zeiss), respectively. The beam power reaching the sample was 31.2 mW/cm^2^ (LI-COR Biosciences, Lincoln, NE, USA) and the amount of energy absorbed by pigment was taken in the account in determination of τ_1/2_ values. The spectrophotometrical monitoring of the lowest energy band decay during irradiation was carried out until a significant degradation of pigment occurred and the kinetic traces could be obtained. All experiments were carried out at ambient temperature in 1 cm quartz cuvettes.

#### 3.2.4. Spectroscopy

The electronic absorption spectra were recorded on a Cary 50 Bio spectrophotometer (Varian, Sydney, Australia). The steady state emission spectra were measured on a Fluorolog Max-P spectrofluorometer (Horiba Jobin Yvon, Kyoto, Japan). The CD spectra were recorded on a J815 spectropolarimeter (JASCO, Tokyo, Japan). All spectra were recorded at ambient temperature. The fluorescence lifetimes were measured on a time-domain Chronos BH fluorometer (ISS, Baltimore, MD, USA) using a picoseconds laser diode (478 nm, pulse duration of 74 ps, output power of 103 mW, frequency of 20 MHz) for the excitation and a H7422P-50 photomultiplier (Hamamatsu, Hamamatsu City, Japan). A milk powder suspension in distilled water was applied as the reference scattering sample. Fitting of theoretical curves to the experimental data was performed using the bundled ISS Vinci software.

## 4. Conclusions

The choice of model chromophores and a thorough analysis of their properties with the use of the same methodology facilitated a clear distinction between various structural factors affecting the photophysics of chlorophyll-related pigments. We have focused on the features which seem most relevant for solar energy conversion in man-made systems. Obviously, the effects of gross structural modifications of these pigments are well documented in the literature. However, the present spectroscopic analyses of model pigments provide a systematic gradation of how specific fine structural changes affect the ground- and excited-state properties of the (B)Chls in question. In particular, we were able to show and explain the effects of the central metal ion and peripheral electron-withdrawing groups (carbonyls). Also, the factors influencing the photostability of the pigments were determined, which is very important for their application as the photoactive components. These new pieces of information may serve as a reference and guidelines for the design of artificial solar energy conversion systems based on the naturally occurring (B)Chls.

In conclusion, chemical modifications may substantially improve properties of (bacterio)chlorophyll-derived pigments in the context of their application for solar energy conversion. The rational alteration of their conservative structural features, such as the macrocyclic π-electron system, CMI, or peripheral substituents, may allow for improvement of their ground- and excited-state parameters or other important properties, in particular the pigment photostability. The latter parameter seems highly relevant for devices performing solar energy conversion as in natural photosynthetic systems the intrinsic photoprotective and regenerative mechanisms serve to cope with overexcitation of the photosynthetic apparatus.

The macrocyclic π-electron system influences pigments’ ground- and excited-states to the greatest extent and hence the modifications of its structure, *e.g*. reduction of pyrrolenine rings, allows for the most significant control over the relevant parameters. The modifications of the π-electron system change the molecular symmetry which determines the energy levels of HOMO and LUMO orbitals and hence governs the essential parameters, including the S_1_ energy, the S_1_ state lifetime and quantum yield. In addition, the S_1_ energies in BChla and BPheo strongly depend on the aggregation state of the pigment.

The CMI may be considered as the second most influential structural feature of (B)Chls. Its modifications only moderately influence the ground-state properties of (B)Chls, but the excited-state parameters (S_1_ state lifetime and quantum yield) are highly CMI-dependent. Importantly, the presence of CMI heavier than Mg^2+^ significantly improves the pigment photostability, however, at the expense of S_1_ state lifetime, which may be critical for singlet energy transfer required to drive photochemical reactions. An area for improvement would be in achieving a better control over quantum yields of the excited states, which would be particularly useful in potential practical applications. In this context, modifications of the peripheral substituents may also be beneficial, especially when considering their influence on pigment aggregation, photostabilities, and redox potentials.

## Supplementary Materials

Supplementary materials can be accessed at: http://www.mdpi.com/1420-3049/19/10/15938/s1.

## Supplementary Files

Supplementary File 1

## References

[1] Zhang S.F., Yang X.D., Numata Y.H., Han L.Y. (2013). Highly efficient dye-sensitized solar cells: Progress and future challenges. Energ. Environ. Sci..

[2] Nocera D.G. (2012). The artificial leaf. Acc. Chem. Res..

[3] Kobuke Y. (2006). Artificial light-harvesting systems by use of metal coordination. Eur. J. Inorg. Chem..

[4] Young K.J., Martini L.A., Milot R.L., Snoeberger R.C., Batista V.S., Schmuttenmaer C.A., Crabtree R.H., Brudvig G.W. (2012). Light-driven water oxidation for solar fuels. Coord. Chem. Rev..

[5] Li Y., Cai Z.-L., Chen M. (2013). Spectroscopic properties of chlorophyll f. J. Phys. Chem. B.

[6] Grimm B., Porra R.J., Rüdiger W., Scheer H. (2006). Chlorophylls and Bacteriochlorophylls.

[7] Scheer H. (1991). Chlorophylls.

[8] Willows R.D., Li Y., Scheer H., Chen M. (2013). Structure of chlorophyllf. Org. Lett..

[9] Orf G.S., Tank M., Vogl K., Niedzwiedzki D.M., Bryant D.A., Blankenship R.E. (2013). Spectroscopic insights into the decreased efficiency of chlorosomes containing bacteriochlorophyll f. BBA-Bioenergetics.

[10] Norris J.R., Raghavan M. (1991). Strategies for mimicking the primary events of bacterial photosynthesis—structure, function, and mechanism. Photochemical Conversion and Storage of Solar Energy.

[11] Henderson B.W., Sumlin A.B., Owczarczak B.L., Dougherty T.J. (1991). Bacteriochlorophyll-a as photosensitizer for photodynamic treatment of transplantable murine tumors. J. Photochem. Photobiol. B.

[12] Fiedor L., Gorman A.A., Hamblett I., Rosenbach-Belkin V., Salomon Y., Scherz A., Tregub I. (1993). A pulsed laser and pulse radiolysis study of amphiphilic chlorophyll derivatives with pdt activity toward malignant melanoma. Photochem. Photobiol..

[13] Rosenbach-Belkin V., Chen L., Fiedor L., Tregub I., Pavlotsky F., Brumfeld V., Salomon Y., Scherz A. (1996). Serine conjugates of chlorophyll and bacteriochlorophyll: Photocytotoxicity *in vitro* and tissue distribution in mice bearing melanoma tumors. Photochem. Photobiol..

[14] Blankenship R.E., Blankenship R.E. (2008). Photosynthetic pigments: Structure and spectroscopy. Molecular Mechanisms of Photosynthesis.

[15] Tamiaki H., Kunieda M., Kadish K.M., Smith K.M., Guilard R. (2011). Photochemistry of chlorophylls and their synthetic analogs. Handbook of Porphyrin Science.

[16] Chen M., Scheer H. (2013). Extending the limits of natural photosynthesis and implications for technical light harvesting. J. Porphyrins Phthalocyanines.

[17] Brancaleon L., Magennis S.W., Samuel I.D.W., Namdas E., Lesar A., Moseley H. (2004). Characterization of the photoproducts of protoporphyrin ix bound to human serum albumin and immunoglobuling. Biophys. Chem..

[18] Weber G., Teale F.W.J. (1957). Determination of the absolute quantum yield of fluorescent solutions. Trans. Faraday Soc..

[19] Fiedor L., Stąsiek M., Myśliwa-Kurdziel B., Strzałka K. (2003). Phytol as one of the determinants of chlorophyll interactions in solution. Photosynth. Res..

[20] Losev A.P., Sagun E.I., Kochubeev G.A., Nichiporovich I.N. (1986). Fluorescence quantum yields, lifetimes, and critical distances for energy transfer for chlorophyll and its pheophytin in solutions. J. Appl. Spectrosc..

[21] Fiedor L., Kania A., Myśliwa-Kurdziel B., Stochel G. (2008). Understanding chlorophylls: Central magnesium and phytyl as structural determinants. Biochim. Biophys. Acta.

[22] Drzewiecka-Matuszek A., Skalna A., Karocki A., Stochel G., Fiedor L. (2005). Effects of heavy central metal on the ground and excited states of chlorophyll. J. Biol. Inorg. Chem..

[23] Pilch M., Dudkowiak A., Jurzyk B., Łukasiewicz J., Susz A., Stochel G., Fiedor L. (2013). Molecular symmetry determines the mechanism of a very efficient ultrafast excitation-to-heat conversion in ni-substituted chlorophylls. Biochim. Biophys. Acta.

[24] Fery-Forgues S., Lavabre D. (1999). Are fluorescence quantum yields so tricky to measure? A demonstration using familiar stationery products. J. Chem. Educ..

[25] Morris J.V., Mahaney M.A., Huber J.R. (1976). Fluorescence quantum yield determinations. 9,10-diphenylanthracene as a reference standard in different solvents. J. Phys. Chem..

[26] Niedzwiedzki D.M., Blankenship R.E. (2010). Singlet and triplet excited state properties of natural chlorophylls and bacteriochlorophylls. Photosynth. Res..

[27] Rosenbach-Belkin V., Fisher J.R.E., Scherz A. (1991). Effect of nonexcitonic interactions among the paired molecules on the qy transition of bacteriochlorophyll dimers—applications to the primary electron-donors p-860 and p-960 in bacterial reaction centers. J. Am. Chem. Soc..

[28] Gouterman M., Dolphin D. (1978). Optical spectra and electronic structure of porphyrins and related rings. The Porphyrins.

[29] Vernon L.P., Seely G.R. (1966). The Chlorophylls.

[30] Gouterman M., Wagniere G.H., Snyder L.C. (1972). Spectra of porphyrins. Part ii. Four orbital model. J. Mol. Spectr..

[31] De Paula J.C., Robblee J.H., Pasternack R.F. (1995). Aggregation of chlorophyll a probed by resonance light scattering spectroscopy. Biophys. J..

[32] Beddard G.S., Porter G. (1976). Concentration quenching in chlorophyll. Nature.

[33] Hartwich G., Fiedor L., Simonin I., Cmiel E., Schäfer W., Noy D., Scherz A., Scheer H. (1998). Metal-substituted bacteriochlorophylls. 1. Preparation and influence of metal and coordination on spectra. J. Am. Chem. Soc..

[34] Küpper H., Dedic R., Svoboda A., Hala J., Kroneck P.M.H. (2002). Kinetics and efficiency of excitation transfer from chlorophylls, their heavy metal-substituted derivatives, and pheophytins to singlet oxygen. Biochim. Biophys. Acta.

[35] Teuchner K., Stiel H., Leupold D., Scherz A., Noy D., Simonin I., Hartwich G., Scheer H. (1997). Fluorescence and the excited state absorption in modified pigments of bacterial photosynthesis. A comparative study of metal-substituted bacteriochlorophylls a. J. Lumin..

[36] Callahan P.M., Cotton T.M. (1987). Assignment of bacteriochlorophyll a ligation state from absorption and resonance raman spectra. J. Am. Chem. Soc..

[37] Noy D., Fiedor L., Hartwich G., Scheer H., Scherz A. (1998). Metal-substituted bacteriochlorophylls. 2. Changes in redox potentials and electronic transition energies are dominated by intramolecular electrostatic interactions. J. Am. Chem. Soc..

[38] Karweik D.H., Winograd N. (1976). Nitrogen charge-distributions in free-base porphyrins, metalloporphyrins, and their reduced analogs observed by x-ray photoelectron-spectroscopy. Inorg. Chem..

[39] Kania A., Pilch M., Rutkowska-Żbik D., Susz A., Heriyanto, Stochel G., Fiedor L. (2014). High-pressure and theoretical studies reveal significant differences in the electronic structure and bonding of magnesium, zinc, and nickel ions in metalloporphyrinoids. Inorg. Chem..

[40] Kania A., Fiedor L. (2006). Steric control of bacteriochlorophyll ligation. J. Am. Chem. Soc..

[41] Sauer K., Smith J.R.L., Schultz A.J. (1966). The dimerization of chlorophyll a, chlorophyll b, and bacteriochlorophyll in solution. J. Am. Chem. Soc..

[42] Fajer J. (2004). Chlorophyll chemistry before and after crystals of photosynthetic reaction centers. Photosynth. Res..

[43] Fiedor J., Fiedor L., Kammhuber N., Scherz A., Scheer H. (2002). Photodynamics of the bacteriochlorophyll-carotenoid system. 1. Influence of central metal, solvent and β-carotene on photobleaching of bacteriochlorophyll derivatives. Photochem. Photobiol..

[44] Watanabe T., Kobayashi M., Scheer H. (1991). Electrochemistry of chlorophylls. Chlorophylls.

[45] Barzda V., Peterman E.J.G., van Grondelle R., van Amerongen H. (1998). The influence of aggregation on triplet formation in light-harvesting chlorophyll a/b pigment-protein complex ii of green plants. Biochemistry.

[46] Brody S.S., Brody M. (1962). Fluorescence properties of aggregated chlorophyll *in vivo* and *in vitro*. Trans. Faraday Soc..

[47] Krieger-Liszkay A. (2005). Singlet oxygen production in photosynthesis. J. Exp. Bot..

[48] Iriyama K., Ogura N., Takamiya A. (1974). A simple method for extraction and partial purification of chlorophyll from plant material, using dioxane. J. Biochem..

[49] Omata T., Murata N. (1983). Preparation of chlorophyll a, chlorophyll b and bacteriochlorophyll a by column chromatography with deae-sepharose CL-6B and sepharose CL-6B. Plant Cell Physiol..

[50] Jones I.D., White R.C., Gibbs E., Denard C.D. (1968). Absorption spectra of copper and zinc complexes of pheophytins and pheophorbides. J. Agric. Food Chem..

