# Supplementary material for: Lessons from Chlorophylls: Modifications of Porphyrinoids Towards Optimized Solar Energy Conversion
Source: Molecules. 2014 Oct 3;19(10):15938–54. doi: 10.3390/molecules191015938 (PMC6271569; doi:10.3390/molecules191015938)
Supplement: Supplementary File 1 [file molecules-19-15938-s001.docx]

**Supplementary Materials**

**Mass spectrometry:** the mass spectrometry analyses were carried out using an ACQUITY TQD mass spectrometer (Waters, USA) with an electrospray positive ionisation method. The spectrometer was coupled with an ACQUITY H-Class UPLC apparatus (Waters, USA), equipped with an ACQUITY UPLC column (BEH C18 1.7μm) (2.1 × 50 mm, Waters, USA). Methanol acidified with 0.1% formic acid was used as eluent.

Table S1. Mass spectrometry analyses of the free base (Pheo) and metal complexes

| **Compound** | **Monoisotopic Mass [u]** | **Measured Mass**  **[*m/z*]** | **Interpretation** | **Highest Peak** |
| --- | --- | --- | --- | --- |
| Chla | 892.53 | 892.71 | Molecular mass | 893.61 |
| Chlb | 906.51 | 906.71 | Molecular mass | 906.71 |
| Bchla | 910.54 | 910.82 | Molecular mass | 926.72 |
| Pheo | 870.56 | 871.52 | Molecular mass | 871.52 |
| BPheo | 888.57 | 888.71 | Molecular mass | 888.71 |
| Zn-Pheo | 932.48 | 948.83 | Oxidized molecule | 948.83 |
| Ni-Pheo | 926.48 | 958.48 | Methanol adduct | 958.48 |
| Pt-Pheo | 1063.51 | 1079.41 | Oxidized molecule | 301.10 |
